# Participatory development of nursing curricula – a Global North–South partnership between Norwegian and Ethiopian universities

**DOI:** 10.1186/s12912-026-04464-8

**Published:** 2026-03-02

**Authors:** Abdallah Abudayya, Tesfaye Hordofa Leta, Åshild Gjellestad, Abebe Abera, Begna Tulu, Asresash Demissie, Temesgen Kabeta, Asnake Ararsa Irenso, Nima Neolene Wesseltoft-Rao, Zada Pajalic

**Affiliations:** 1 Institute of Nursing, Faculty of Health Science, VID Specialized University, P.b. 184 Vinderen, , Oslo, NO-0319 Norway; 2 Department of Nursing and Health Promotion, Faculty of Health Sciences, Oslo Metropolitan University, Oslo, Norway; 3 Department of Nursing, Institute of Health Jimma University, Jimma, Ethiopia; 4 Department of Medical Laboratory Sciences, College of Medicine and Health Sciences, Ambo University, Ambo, Ethiopia; 5 Nursing and Health Sciences, Faculty of Medicine, Monash University, Clayton, Australia; 6 Faculty of Health and Social Sciences, University of South-Eastern Norway (USN), Drammen, Norway

**Keywords:** Global North-South collaboration, Internationalization, Capacity building, Higher education, Student active learning, Global health nursing, Participatory action research

## Abstract

Transnational academic collaboration, particularly global North-South, is increasingly recognized as a critical strategy for enhancing educational quality, equity, and relevance in global health and nursing education. This study aimed to describe the participatory process of developing and revising two joint Master’s level course drafts, Global Health Nursing and Participatory Action Research in Health Care, and to explore the feasibility of integrating these courses into the existing curricula of partner universities in Norway and Ethiopia. A qualitative descriptive design was employed, grounded in a participatory approach. Data were collected during a structured workshop using the Story Dialogue Method (SDM), which engaged 20 academic participants from both countries in reflective discussions and collaborative revisions of course drafts. Thematic insights were drawn from dialogue notes and synthesized through group-based reflection and analysis. The findings revealed both opportunities and challenges in joint curriculum development. The collaborative process fostered pedagogical innovation, mutual capacity building, and student-centered-learning approaches. However, integration into existing programs, especially in the Ethiopian context, was hindered by less flexible institutional structures, digital infrastructure limitations, and the ambitious scope of learning outcomes relative to course design. Thus, discussions emphasized the need for clearer course content alignment with credit allocation and institutional capacities. This study underscored the potential of equitable and participatory academic partnerships to co-create contextually relevant and globally informed curricula. For such initiatives to be sustainable and impactful, future efforts should include broader stakeholder engagement, realistic curricular planning, and strategic measures to bridge digital and institutional divides.

## Clinical trial number

Not applicable.

## Highlights

Participatory Story Dialogue Method (SDM) was used to collaboratively develop and revise joint Master’s level nursing courses between Norwegian and Ethiopian universities.

Two digital, co-created courses—Global Health Nursing and Participatory Action Research in Health Care—were developed using student-centered, problem-based learning approaches.

Curricula were adapted to local contexts and health challenges, promoting culturally responsive and globally relevant nursing education.

Digital infrastructure gaps and institutional rigidity in Ethiopian universities posed major challenges to course integration and delivery.

Equitable academic partnership fostered mutual capacity building, shared teaching responsibilities, and promoted gender equity in higher education.

## Introduction

Universities typically have three important responsibilities: education, research collaboration, and community engagement [1, 2]. These functions operate within the context of society at local, national and international levels [3, 4]. Knowledge transfer and collaboration are vital approaches for universities globally [5, 6]. The goal of globalizing university studies is to enhance both teaching [7] and learning, create opportunities for international students [8], and equip staff with the necessary skills to address current and future challenges in the job market [9, 10]. The primary purpose of the university’s mobilization is to offer the best possible higher education qualifications [10, 11]. However, this aspiration faces challenges, particularly for students from the global south seeking to study abroad, as visa requirements can pose an obstacle [2]. The importance of international cooperation [12, 13] has been more thoroughly validated during the prolonged global crisis brought on by the COVID-19 pandemic. The pandemic highlighted the reality that we confront shared challenges that can only be effectively addressed through collective efforts [12].

## Background

Academic discourses critically engage with dominant models of international relations and development, particularly within the Global North–South context of higher education. Real-world examples of collaborative initiatives between institutions in the Global North and South illustrate how such partnerships are negotiated, why they succeed or fail, and what implications they have for equity, knowledge production, and sustainable development [14, 15]. Critics argue that the traditional ‘North-South’ educational and research collaborations often perpetuate a neo-colonial dynamic where knowledge and resources flow predominantly from wealthier ‘Northern’ countries to ‘Southern’ counterparts, potentially undermining the agency and development of institutions in the Global South [16, 17]. This critique calls for a rethinking of ‘capacity building’ to move away from a donor-recipient model towards a more equitable partnership that respects and utilizes the unique contributions of all involved [18]. These partnerships are challenged by profound disparities in socioeconomic conditions, cultural environments, infrastructure, and institutional capacities [19]. Such differences often skew the collaborative landscape, with institutions from the North typically dominating discourse and decision-making power [17]. Transforming North–South partnerships involves addressing power imbalances through equitable and reciprocal collaboration. This means recognizing and valuing diverse forms of knowledge, particularly those from the Global South, ensuring shared decision-making, and promoting co-creation rather than one-sided knowledge transfer [20]. True partnership, in this context, implies full and equitable participation of Southern partners, who contribute essential local knowledge and legitimacy to collaborative efforts. This involves joint decision-making, mutual trust, transparent information sharing, equitable resource allocation, and shared responsibilities throughout the planning, implementation, and evaluation of projects (Horner & Hulme, 2019).

Challenges to these partnerships often include infrastructural limitations in the South, such as unreliable internet connectivity or limited research infrastructure, which can hinder communication, data sharing, and participation in joint activities [21]. Additionally, mismatches in institutional priorities, where Northern partners may emphasize research outputs and publications, while Southern partners may prioritize capacity building or community engagement, can lead to misaligned goals and uneven benefits [22]. These problems are exacerbated by a persistent one-size-fits-all approach that disregards the socio-political, cultural, and economic contexts of Southern institutions, reducing the relevance and impact of the collaboration. Furthermore, the typically short duration of such partnerships often prevents the establishment of trust, continuity, and institutional learning, ultimately compromising the sustainability and long-term effectiveness of the initiatives [23].

The Enhancing Public Health Capacity through Academic Network and Mobility (EPHAM) project under the Norwegian Partnership Program for Global Academic Cooperation (NORPART) illustrates the operationalization of these principles. This initiative between one Norwegian and two Ethiopian universities is oriented towards Ethiopia’s high disease burden and towards Norway’s future with a multiethnic and ageing population and a shortage of healthcare workers [24, 25]. Currently, only a quarter of the necessary healthcare workforce is in place to adequately serve the population in Norway [26]. For Norway, it is valuable to explore how nursing and health services are organized within entirely different system perspectives and healthcare structures. A comprehensive understanding of cultural competencies is essential for the successful implementation of the initiatives. Gaining insight into how nursing is practiced across hospitals, communities, and families in other contexts can help inform and improve the delivery of nursing services in Norway.

The rapid expansion of higher education institutions in Ethiopia has further eroded the quality of both scientific staff and healthcare services [27, 28]. Thus, the EPHAM-project specifically aims to enhance the capacity of clinical nursing and public health master’s programs, increase the mobility of teachers and students between partner institutions, develop joint courses in Participatory Action Research (PAR) in Health Care and Global Health Nursing, and improve qualifications for female academics to minimize gender disparities. These goals were aimed to be pursued through the development of joint courses, collaborative teaching, comprehensive guidance, and joint supervision of students during both clinical internships and thesis writing, and will benefit both VID and the universities in Ethiopia in the future.

The project further aims to foster mutual student and teacher mobility between Norway and Ethiopia, facilitated by joint teaching, guest lectures, development of e-learning materials, and active participation in clinical and community-based practices. Thus, the project’s activities not only underline the collaborative effort but also integrate it into the long-term strategic objectives of the participating Norwegian institution. In EPHAM, we are continuously learning and documenting what actions to take during active project phases and after the project concludes. These include adapting courses to current and emerging health challenges. This includes continuous discussions, feedback, collaborative revisions, and a thorough approach to developing the curriculum.

Thus, the present study aimed to describe the participatory process of developing and revising two joint Master’s level course drafts, Global Health Nursing and Participatory Action Research in Health Care, and to explore the feasibility of integrating these courses into the existing curricula of partner universities in Norway and Ethiopia.

## Methods

### Design

The present study utilized a qualitative descriptive approach [29, 30] to understand and describe the process of revising course drafts. This approach was embedded within the participatory design framework [31], emphasizing collaboration with participants throughout the research process. The data were collected during a workshop using the Story Dialogue Method (SDM) [9, 32, 33] to facilitate narrative sharing and discussion.

### Study context

The first kick-off EPHAM project team meeting in May 2022 focused on planning two Master’s syllabi to be developed within the project’s framework. These courses (*Global Health Nursing and PAR in Health Care*) would have 5 European Credit Transfer Systems (ECTS) each and were offered fully digitally using the web-based Canvas learning platform. The first admission to these courses was conducted during the spring semester of 2023. Firstly, PAR in Health Care (5 ECTS credits), which aimed to explore ways to include practitioners, patients, decision-makers, and the community in the research process. Secondly, Global Healthcare Nursing (5 ECTS credits), which focused on existing public health priorities and adaptable to local realities to ensure relevance and impact. The courses were tailored to fit the local needs and challenges faced by students across locations by engaging with local health professionals and stakeholders during the syllabus design process. The pedagogical methods that dominated the subjects were to be student-centered and problem-based learning [34]. To measure student satisfaction and knowledge retention, we sat up feedback tools like surveys or assessments at the end of courses. Making these results publicly available would enhance transparency and demonstrate the initiative’s value. The teaching would be in English and would be completed with digital solutions by co-authors’ expert contributions. Both course plans were quality-checked by an advisor in the study section before being discussed at a Faculty of Health Sciences leadership meeting at VID. Furthermore, the courses were assessed by the Internationalization Section. The ambition was to offer the courses once per year to Nursing and public health students at the partner universities in Ethiopia, the Master’s program in clinical nursing (MAKS) at VID, and external applicants who met the requirements to study at the Master’s level in the health sciences.

### Participants

All participants in the workshop (*n* = 20) from Norway (*n* = 12) and Ethiopia (*n* = 8) were included in this study. The age of the participants ranged from 30 to 67 years and > 75% of the participants were male.

### Data collection and analysis

Data were generated through a structured workshop using the Story Dialogue Method (SDM), a participatory approach that integrates collective reflection, dialogue, and shared analysis [32, 33]. The SDM workshop was facilitated by the last author (ZP) and involved academic staff from Norwegian and Ethiopian partner institutions. Participants were divided into mixed groups consisting of representatives from different universities. Four groups participated physically, while one group participated digitally via the Teams platform. Each group selected a moderator responsible for facilitating dialogue and a secretary responsible for documenting the discussions. The workshop was a dedicated SDM session organized specifically for academic staff involved in the EPHAM project from the Norwegian and Ethiopian partner institutions. Its purpose was to review and revise the two joint Master’s-level course drafts. There was one structured workshop focused solely on this activity.

The trigger for the SDM process was the systematic discussion and revision of draft course descriptions for Global Health Nursing and Participatory Action Research in Health Care. The workshop followed the predefined SDM steps, including reflection circles, structured dialogue guided by “what,” “why,” “so what,” and “now what” questions, story recording, and the creation of insight cards [32, 33].These steps were used to stimulate reflection on similarities, differences, challenges, and opportunities related to the course drafts. Written notes produced by the group secretaries during each SDM step constituted the primary data material. The insight cards, developed collaboratively at the end of the workshop, summarized the most important issues identified during the dialogue.

Data analysis was conducted in line with the participatory principles of SDM, where data generation and analysis occur simultaneously through dialogue and reflection [33]. During the workshop, participants jointly identified challenges, needs, and opportunities related to course structure, learning outcomes, feasibility, and implementation. These shared reflections represented the first level of analysis.

Following the workshop, all written material, including group discussion notes and insight cards, was reviewed iteratively by the authors. The material was read repeatedly to identify meaning units related to course design, learning outcomes, workload and credit allocation, digital delivery, integration into existing master’s program, and collaborative teaching. The meaning units were compared across groups and clustered into broader thematic categories reflecting recurring patterns in the data. Through iterative discussion and consensus among the authors, the final themes were refined and agreed upon. These themes constitute the basis for the thematic presentation of the results, consistent with previous SDM-based studies [9, 33]. All SDM steps are illustrated in Table 1.

**Table 1:** Structured SDM Guiding Questions with Time Allocation

SDM Step	Guiding Question Category	Explicit Questions Used in the Workshop	Approximate Time
Step 1	Introduction	• Introduction to SDM procedure • Presentation of workshop objectives • Participant introductions	15 min
Step 2	WHAT (Descriptive questions)	• What is the identified need or challenge in the course drafts? • What aspects of the draft were successful? • What similarities or differences exist compared to current curricula? • What educational prerequisites or cross-national challenges may affect implementation?	15 min
Step 3	WHY (Explanatory questions)	• Why might these courses work in your institutional context? • Why might they not work? • Why are certain learning outcomes perceived as ambitious or unclear? • How does your reasoning align with the project’s goals?	Part of 70 min structured dialogue
	SO WHAT (Analytical/Synthesis questions)	• What have we learned from this discussion? • What remains a key challenge for feasibility and integration? • What are the implications for curriculum structure, workload, and credit allocation? • How can these courses benefit Master’s students and faculty?	
	NOW WHAT (Action-oriented questions)	• What concrete revisions are required to improve transparency and clarity? • What actions are necessary to align learning outcomes with 5 ECTS credits? • How can integration into existing programs be facilitated? • How can colleagues be supported in implementing and teaching the courses?	
Step 4	Story Record	• Sharing of reflections across groups	10 min
Step 5	Creation of Insight Cards	• Identification of 4–8 key insights summarizing discussions related to the theme	10 min

### Ethical considerations

Ethical approval for this study was granted by the Norwegian Agency for Shared Services in Education and Research (SIKT), under reference number 793,452. The work of NSD/Sikt was closely aligned with the principles of the Helsinki Declaration, providing guidance to researchers in planning and conducting studies that are fair, ethical, and legally compliant. In this way, they served as a practical support system to ensure that international research ethics standards are upheld in Norway.

All participants in the workshop were anonymized to protect their privacy and informed of their right to withdraw from the study at any stage without any consequences. Written informed consent was obtained from all participants prior to participation. All data were stored securely on the university’s research server (REDACTED) in accordance with confidentiality and data protection regulations.

## Results

The results present the findings from the Story Dialogue Method (SDM) workshop, focusing on participants’ shared reflections on the development and feasibility of the two joint master’s-level courses. Analysis of the workshop discussions revealed several interrelated themes that captured both perceived strengths and challenges related to (i) course structure, content, and pedagogical design; (ii) ambition and clarity of intended learning outcomes; (iii) workload, credit allocation, and self-directed learning; (iv) feasibility of integration into existing master’s programs; and (v) collaborative teaching and shared academic responsibility. The themes below reflect patterns that emerged across the different stages of the SDM process.

### Course structure, content, and pedagogical design

The course drafts have been written in the existing course template at a Norwegian university. The template contains mandatory headings such as course content, main subjects, and learning objectives, divided into knowledge, skills, and general competence. Furthermore, rubrics focus on work and teaching methods, collective activities, attendance, and assessment information. Again, there was a heading that included, among other things, information on the number of ECTS offered as online courses and the minimum eligibility requirements for applying to these courses. Course literature was presented in the course outline. The PAR in Health Care course mainly focused on understanding what it meant to use the PAR method. PAR in healthcare addressed practical issues in improvement and development based on understanding the social context and involving participants based on their conditions. Using PAR enabled the exploration of areas for improvement in practice and promoted the joint development of all involved. The Global Health Nursing course primarily focused on health systems, health promotion and prevention, global nursing in specifically challenging situations, and global nursing roles and responsibilities related to Sustainable Development Goals. Throughout the course, different perspectives on global health challenges and on developing approaches to potential solutions could be analyzed. Working and teaching methods in classes include digital (synchrony and asynchrony) lectures and seminars in smaller groups (using break-out rooms on Zoom), presentations and student-active learning group work, such as writing papers. The teaching would be based on a problem-oriented and collaborative approach to learning where the assigned tasks provide opportunities for the student to take active responsibility for their learning. There was also an emphasis on equality in discussions between students and lecturers. To encourage students’ learning and creativity, the form of examination is to be an opportunity to choose between a product in the form of a written essay, a podcast, or a video presentation.

In the first step of the SDM, several questions were asked, and problems and needs were identified (Table 1). Following this initial exploration, participants shared their immediate reflections on the draft courses, which were generally positive, describing the content as engaging and the structure as clear. However, despite this overall positive assessment, participants emphasized the need for greater clarity, as some content and topics were perceived as not sufficiently straightforward. Consequently, a consensus emerged that further work was needed to specify and refine the subject areas. For participants from Ethiopia, this curriculum structure was new and differed considerably from how course plans are traditionally organized in their context.

### Ambition and clarity of intended learning outcomes

Initial reflections on the course suggested that the intended learning outcomes may be overly ambitious, broad, and optimistic, particularly given the scope and time constraints of a 5 ECTS master’s-level course.

Questions about possible challenges led to discussions about the distinction between knowledge, skills, and general knowledge in the course drafts. Those formal learning outcomes were perceived as unclear in content and curricula. Everyone agreed upon the importance of being transparent about the content and main subject, and some concrete proposals have been discussed.

### Workload, credit allocation, and self-directed learning

Furthermore, while discussing “so what questions” and the remaining challenges, the participants talked about the practical implementation of courses. The first reflection was on how these two courses could be integrated into the existing Master’s programs. Another key aspect discussed was the formal resources available for the courses. Each course, worth 5 ECTS, included a total of 135 h, with 15 h allocated to tuition and other learning activities. At the same time, 120 h were designed for self-study. These courses were built on the education platform Canvas. For those unfamiliar with Canvas, there was an introduction or pre-course Canvas training, along with ongoing support. The lectures covering the most critical themes were delivered via synchronous Zoom sessions or as pre-recorded asynchronous lectures. Seminars emphasized student-active methods and were held synchronously on Zoom. Digital courses required detailed planning and a continuous flow of information for students, which in turn, meant students needed to take responsibility for their learning. Ethiopian participants highlighted that the digital setup demands a high level of self-responsibility and involves relatively few teacher-led hours.

### Feasibility of integration into existing master’s programs

The participants from Norway had the opportunity to integrate these courses into the ongoing Master’s program in clinical nursing, whereas the participants from Ethiopia stated that their Master’s programs do not have the flexibility to include these two courses. In terms of teaching methods, Ethiopian participants mentioned potential challenges with unstable electricity and limited internet access while these digital courses are in progress.

### Collaborative teaching and shared academic responsibility

Discussions about “now what” questions, such as how we identify opportunities with this curriculum and how we support colleagues when involving them, raised issues regarding teaching and the participation of everyone involved. The discussion highlighted that the project group has a wide range of expertise in research and pedagogy. Everyone agreed that these skills should be integrated into education. Since the courses will be created and integrated at a Norwegian institution, there will also be course hubs sharing responsibility for exams and university ECTS credits. These courses were launched for the first time in spring 2023 as planned. After the initial round, feedback from students and teachers served as a basis for further adjustments. Ultimately, the most important insights or keywords were summarized in seven insight cards.

**Insight the cards resulted in**:

1. Courses have the potential to be further specified.
2. The content of the course plans must be adapted to the number of study’s ECTS credits.
3. The digital design of the course can be challenging for Ethiopian partners due to the infrastructure.
4. The courses must be integrated into the existing Master’s program.
5. Teaching staff from both countries will make it possible to share teaching tasks.
6. Student active methods might be news to Ethiopian students.
7. The importance that students at Master’s level understand the level of self-responsibility to follow instructions and become prepared for seminars.

## Discussion

This study examined the participatory development and feasibility of two joint Master’s-level courses through a structured SDM workshop. The discussion is organized around the five main themes identified in the results. These themes collectively highlight both the opportunities and the structural limitations of transnational curriculum co-creation within a Global North–South partnership.

### Course structure, content, and pedagogical design

Developing joint courses for Global Health Nursing and PAR in Healthcare represented a significant effort to create a transdisciplinary, evidence-based educational framework. In this discussion, we examined key themes from an SDM workshop, highlighting important aspects such as course structure, content, teaching methods, integration into existing programs, digital challenges, and the collaborative role of instructors. Recent research articles [13] emphasized the importance of program integration and alignment in higher education, underscoring their relevance for incorporating new courses into established curricula. Nguyen et al. addressed digital challenges in education, stressing the need to close the digital divide to ensure equitable access to learning opportunities [35]. Two other studies promoted collaborative teaching approaches, emphasizing the benefits of combining diverse expertise to enrich the learning experience [36, 37]. Furthermore, Zakrajsek, T.D. (2023) explores effective teaching strategies, underlining the importance of well-designed courses to guide and enhance the learning process [38]. Additional studies highlight the effectiveness of active learning methods, aligning with our focus on student engagement and teaching strategies [10, 39, 40]. Lastly, another study emphasizes ongoing assessment and evaluation in educational programs, aligning with our consideration of future adjustments and assessment in these joint courses [41].

### Ambition and clarity of intended learning outcomes

The present study indicates that the themes of course structure and course content were essential in ensuring compelling learning experiences. The favorable reception of the initial draft of the course underscored the importance of a well-organized, clear course design. However, participants in the discussions consistently emphasized the need for more precise specification and alignment of course content, particularly in relation to the expected learning outcomes at the master’s level and the appropriate allocation of credit hours. The intended learning outcomes were perceived as overly ambitious in scope, encompassing a breadth and depth of knowledge, skills, and general competence that exceeded what could realistically be achieved within a single 5 ECTS course. Several outcomes implicitly assumed levels of theoretical mastery and applied competence more consistent with larger or multi-course units. This perspective aligns with contemporary research [42], emphasizing the critical role of well-structured courses in providing a clear roadmap for effective learning [43].

Biggs et al. (2022) emphasized the significance of aligning course content with intended learning outcomes and assessment criteria through the principle of constructive alignment, which demanded coherence between what students were expected to learn, how they learn, and how learning was assessed [44]. From this perspective, overly broad learning outcomes could weaken alignment and compromise assessment validity. Additionally, their concept of constructive alignment underlined the need for a coherent relationship between teaching, learning activities, and assessment. Moreover, Deeley and Bovill emphasized the value of co-creating learning outcomes and course content with students, contributing to a more learner-centered approach and fostering a sense of ownership and engagement [7].

### Workload, credit allocation, and self-directed learning

The present study emphasized that the EPHAM-project would contribute to active teaching methods and to enhancing student engagement, which were central to fostering effective learning environments. Freeman et al. stressed that the focus on incorporating active learning methods such as seminars, presentations, and student-active papers aligned with recent research emphasizing the positive impact of student engagement and participation on enhancing learning outcomes [45]. Furthermore, Zakrajsek & Nilson and Seidel & Tanner highlighted that the flexibility observed in examination formats reflected an awareness of students’ diverse learning styles and preferences, thereby promoting their creativity and critical thinking in the learning process [38, 46].

### Feasibility of integration into existing master’s programs

The findings demonstrated that integrating the courses into existing master’s programs, particularly in Ethiopia, was constrained by institutional rigidity and limited curricular flexibility. These challenges aligned with contemporary studies emphasizing alignment and flexibility when incorporating new techniques into well-established courses [47, 48]. The integration process was crucial for ensuring students’ educationally cohesive and comprehensive experience. Yob et al. examined the challenges and strategies associated with effectively integrating new courses into existing academic programs. The study stressed the significance of aligning the course content with the overarching program objectives, aiming for a whole incorporation that enhances curriculum coherence and enriches the educational journey [47].

### Collaborative teaching and shared academic responsibility

The present study underscored the urgency of addressing digital infrastructural and competency-based challenges in the Ethiopian educational context. By doing so, we endeavored to bridge the digital divide and extend educational advantages to every student, fostering inclusivity and enhancing the overall learning experience. The result of this study aligned with contemporary research that emphasized the urgent need to bridge the digital divide and ensure equal access to educational benefits, particularly in regions facing digital disparities [49, 50].

Addressing these challenges was crucial for guaranteeing that educational opportunities were accessible to all students, regardless of technological circumstances [51], identifying variations in internet skills and usage patterns among different demographics, and shedding light on the digital divide that affects individuals worldwide. In the specific Ethiopian context, Alemu examined the digital range and its implications for educational equity, highlighting the necessity of tailored strategies to address this gap [49].

This study highlighted that collaborative teaching and continuous evaluation strengthen educational quality by integrating the expertise of teaching staff from both countries. Although there were various obstacles in the process, especially regarding the outline and recording of lecture time frames and synchronous attendance, the overall result was believed to have been enriched through the collaborative process [39]. This approach aligned with contemporary research advocating collaborative teaching methods that capitalized on educators’ diverse strengths [52]. As an integral part of this theme, cooperative learning strategies were recognized for their potential to enrich the learning experience, fostering critical thinking and problem-solving skills [4].

Castañeda et al. highlighted the value of interdisciplinary collaboration in teaching, emphasizing how it enhanced the educational experience by integrating varied expertise and perspectives [52]. Clark and Kenaley reinforced this notion by demonstrating that collaborative learning strategies contributed to developing critical thinking and problem-solving skills [4]. The planned evaluation process following the initial course implementation aligned with best practices in educational assessment and evaluation [41]. This planned evaluation, informed by feedback and outcomes, was crucial for continuous improvement and the development of effective educational programs.

### Strengths of the study

A key strength of this study lies in its participatory and collaborative design, which actively involved academic staff from both Norwegian and Ethiopian partner institutions. This approach ensured mutual engagement and contextual relevance, thereby promoting equitable knowledge exchange and joint ownership of the curriculum development process. The use of the *Story Dialogue Method (SDM)* provided a structured yet flexible qualitative framework that facilitated reflective dialogue, co-construction of knowledge, and contextually grounded insights into the course development process.

Furthermore, the study demonstrated a commitment to pedagogical innovation, incorporating student-centered and problem-based learning strategies as well as flexible forms of assessment. These methods align with contemporary trends in higher education aimed at enhancing student engagement and learning outcomes. Additionally, the project emphasized the potential of North–South academic collaborations to foster capacity building, support gender equity by prioritizing the advancement of female academics, and address globally relevant health challenges through localized educational frameworks.

The inclusion of digital learning platforms (e.g., Canvas) and synchronous/asynchronous teaching methods reflects a modern and adaptable teaching approach. The initiative also underscores the bidirectional benefit of such partnerships, as both Norwegian and Ethiopian institutions stood to gain pedagogically, culturally, and institutionally from the collaboration.

### Limitations of the study

Despite its strengths, the study has several limitations that should be acknowledged. First, the scope of participant involvement was limited to academic staff directly affiliated with the EPHAM project. The absence of student perspectives represents a notable limitation, as students are the primary recipients of the curriculum and are directly affected by course workload, learning outcomes, assessment demands, and pedagogical approaches. Excluding critical stakeholders such as students, university administrators (e.g., registrars, program directors), and pedagogical experts external to the project. Their absence may have restricted the breadth and depth of the evaluation, particularly concerning the practical implementation and institutional sustainability of the courses.

Second, the generalizability of the findings is limited, as the study focused on a specific partnership between selected institutions in Norway and Ethiopia. The challenges and solutions identified may not fully apply to other Global North–South collaborations with different cultural, institutional, or infrastructural contexts.

Finally, the generalizability of the findings is limited. The results emerged from a specific Global North–South collaboration between Norwegian and Ethiopian universities, shaped by particular institutional arrangements, resource conditions, and partnership histories. While several insights may resonate with broader discussions on transnational curriculum development, the findings should not be assumed to be directly transferable to all Global North–South academic collaborations. Differences in governance structures, digital infrastructure, academic cultures, and policy environments may lead to substantially different experiences and outcomes in other settings.

### Implications for future research

This study offers important groundwork for understanding participatory curriculum development in cross-national academic partnerships; however, it also opens several avenues for further inquiry. Future research should include the perspectives of additional stakeholders, particularly students, university administrators, and external pedagogical experts. Their insights would offer a more comprehensive understanding of the feasibility, relevance, and institutional support mechanisms necessary for sustainable curriculum integration.

Given the identified challenges related to digital infrastructure and online pedagogy, further studies are needed to investigate how technological limitations in low-income countries affect student engagement, learning outcomes, and instructional delivery. Comparative studies between digital and in-person or hybrid modalities in similar contexts could provide practical guidance for adapting teaching strategies to infrastructural realities.

Moreover, there is a need to evaluate the long-term impact of joint curricula on student competencies, faculty development, and institutional capacity. Longitudinal studies tracking the academic and professional trajectories of students who complete such courses could yield valuable insights into the effectiveness and global applicability of transdisciplinary education.

Finally, future research should explore models of equitable Global North–South academic partnerships, focusing on decision-making structures, power dynamics, and co-authorship practices. This would contribute to the growing discourse on decolonizing knowledge production and fostering genuinely reciprocal collaborations in global higher education.

## Conclusion

This study examined the participatory development of two joint Master’s level courses, Global Health Nursing and Participatory Action Research in Health Care, within the framework of a Global North–South collaboration between Norwegian and Ethiopian universities. Using a qualitative descriptive design and the Story Dialogue Method, the research captured the co-creative and reflective process of designing context-sensitive curricula and assessing their integration within existing academic programs.

The findings illustrated both the potential and the complexity of transnational curriculum development. While the collaboration enabled pedagogical innovation, capacity building, and the contextualization of global health education, it also revealed significant challenges, particularly in relation to institutional flexibility, digital infrastructure, and the scope of learning outcomes within limited credit allocations.

The study emphasized the critical role of equitable, reciprocal partnerships in advancing global education, moving beyond traditional hierarchical models. Collaborative curriculum initiatives, when rooted in mutual respect and shared expertise, could foster active learning, transdisciplinary perspectives, and culturally responsive pedagogy. However, their long-term success depends on addressing structural barriers, ensuring meaningful stakeholder involvement, and aligning educational objectives with institutional and regional capabilities.

This research contributed to the growing body of knowledge on global academic cooperation and offered practical insights for institutions seeking to develop joint curricula that are both globally relevant and locally grounded.

## Data Availability

The data generated and analyzed during this study are not publicly available due to privacy and confidentiality agreements with participants. However, de-identified summaries of the insights and workshop materials may be available from the corresponding author upon reasonable request.

## Declarations

### Declaration of the use of AI

All research processes were conducted and documented by the authors. AI was not used in the design, data collection, analysis, or writing of this study.
